# Metabolic biomarkers differentiate extrapulmonary tuberculosis from pulmonary TB and non-TB pleural effusions

**DOI:** 10.3389/fmed.2026.1793040

**Published:** 2026-06-11

**Authors:** Holly May-Lewis, Michael James Perret, Ye Xu, Roberto Stefan Almeida Ribeiro, Raquel da Silva Correa, Thiago Thomaz Mafort, Ana Paula Santos, Rogério Rufino, Luciana Rodrigues, Khushboo Borah Slater

**Affiliations:** 1 School of Biosciences, University of Surrey, Guildford, United Kingdom; 2 Department of Biosciences, Faculty of Health and Life Sciences, University of Exeter, Exeter, United Kingdom; 3 Department of Clinical Laboratory, Shanghai Ninth People’s Hospital, Shanghai Jiao Tong University School of Medicine, Shanghai, China; 4 Department of Pathology and Laboratories, Laboratory of Immunopathology, Medical Sciences Faculty (FCM), Rio De Janeiro State University (UERJ), Rio de Janeiro, Brazil; 5 Department of Pulmonary Care, Pedro Ernesto University Hospital (HUPE), UERJ, Rio de Janeiro, Brazil

**Keywords:** biomarker, extrapulmonary, metabolomics, *Mycobacterium tuberculosis*, pleural tuberculosis, tuberculosis

## Abstract

Tuberculosis (TB) is one of the leading causes of mortality in humans with clinical manifestations as pulmonary and extrapulmonary forms. TB pleural effusion (PITB) is one of the main forms of extrapulmonary TB, which is challenging to diagnose due to the overlapping clinical symptoms with other non-TB pleural effusions and poses severe complications in the treatment and management of disease. New biomarkers are urgently needed to improve the diagnostic accuracy for PlTB. Here we applied high-throughput targeted liquid chromatography-mass spectrometry (LC-MS/MS) metabolomics using a 96-well plate format to profile metabolic signatures in serum and pleural fluid of PlTB patients. We identified taurine, glycine, tryptophan and kynurenine as the top metabolites for differentiation of PlTB from pulmonary TB and non-TB pleural effusions. Our work provides a new metabolomic assay for biomarker discovery and highlights metabolic signatures with potential to transform the diagnosis of PITB.

## Introduction

Tuberculosis (TB) remains one of the world’s leading causes of death from a single infectious agent despite being a preventable and curable disease (1). *Mycobacterium tuberculosis* (Mtb) causes pulmonary and extrapulmonary TB in humans and is responsible for more than one million deaths every year (1, 2). TB pleural effusion (PlTB) is one of the most common forms of extrapulmonary TB accounting for upto 30% incidences. PlTB is characterized by the accumulation of fluid in the pleural space due to infection with Mtb and host inflammatory reactions to its antigens (3, 4). The prevalence of PlTB is high in endemic regions, and there are growing concerns of drug-resistant cases (3, 5). PlTB presents with a range of clinical features including benign effusions, pleural thickening and empyema resulting in associated complications, lung damage and mortality (3, 4). The clinical signs of PlTB overlap with other non-TB exudative etiologies that lead to pleural effusions such as malignant pleural effusions (MPEs), autoimmune diseases and parapneumonic pleural effusion (PPE). Therefore, accurate clinical diagnosis of pleural TB is challenging (3, 6). Early and accurate diagnosis is needed to manage PlTB and improve treatment outcomes.

The gold standard diagnostic techniques for PITB such as isolation/culture and Ziehl-Neelsen staining for Mtb identification are limited by low sensitivity. Although pleural fluid GeneXpert MTB/Rif (nucleic acid amplification test) provides rapid results, its sensitivity remains limited (7). Additionally, biopsies are time consuming and are invasive procedures with risk of complications (6). Biomarker analysis provides an alternative technique which is inexpensive, non-invasive and sensitive for diagnosis of PlTB (6). Adenosine deaminase (ADA), lactate dehydrogenase (LDH), interferon-gamma (IFN-γ) are commonly used biomarkers for clinical identification. However, the levels of these markers can vary between individuals, which limits the sensitivity and specificity in distinguishing between PlTB and MPEs, leading to delayed treatment or misdiagnosis (4, 8, 9). Therefore, identification of novel biomarkers is an important research area for the development of improved diagnostic tools for PlTB.

Metabolite profiling is a promising technique for screening new candidates and identifying disease-specific markers. Although limited attempts have been made to differentiate between PlTB from healthy individuals and MPEs using metabolite profiling (8–10), no investigation has examined the metabolic markers distinguishing PlTB from pulmonary TB (PTB). Here we conducted high-throughput, targeted metabolomics using liquid chromatography-mass spectrometry (LC-MS/MS) in a 96-well plate format to profile metabolites in pleural fluid and serum from patients with PITB. In this pilot study, we analyzed metabolic profiles from a cohort of 58 individuals to identify signatures that distinguish PlTB from PTB, and non-TB pleural effusions. Our findings reveal novel metabolic signatures uniquely associated with PlTB highlighting their potential utility for precise clinical differentiation from PTB and non-TB pleural effusions.

## Methods

### Participants and sample collection

This study used samples collected as part of a previously published investigation (5), conducted under a protocol approved by the Biomedical Research Ethics Committee of Pedro Ernesto University Hospital, Rio de Janeiro State University (HUPE/UERJ; approval number 1.100.772). All participants provided written informed consent, and all biological specimens were fully anonymized before processing to ensure participant confidentiality. The study was conducted in accordance with the principles of the Declaration of Helsinki.

Samples preserved at -80 °C were obtained from patients aged ≥ 18 years of both sexes (Table 1). A total of 58 samples were analyzed, comprising HC (healthy controls without TB or other diseases), PTB (patients with radiological and/or microbiological confirmation of pulmonary TB), PITB (patients with pleural effusion supported by microbiological confirmation, granulomatous inflammation in pleural tissue, and/or ADA levels ≥ 40 IU/L), and OD (patients with malignant pleural effusions classified as other diseases). Exclusion criteria included pregnancy, age ≤ 18 years, HIV positive individuals and refusal to provide informed consent. Blood samples were collected from all participants via venipuncture. Patients in the PlTB and OD groups presented with unilateral or bilateral pleural effusions and fulfilled established clinical criteria for thoracentesis. Exudative effusions were classified according to Light’s criteria (11). Pleural fluid samples were obtained through ultrasound-guided thoracentesis performed by a certified pulmonologist to ensure accuracy and safety. Following collection, all samples were centrifuged at 850 × *g* to remove cellular debris, and the resulting serum and pleural fluid supernatants were aliquoted and stored at -80 °C until further analysis to preserve metabolite integrity. Serum samples were analyzed for HC, PTB, PlTB, and OD groups, whereas pleural fluid samples were analyzed specifically for PlTB and OD groups.

### Study population and diagnostic criteria

Diagnoses were established by specialist physicians at Pedro Ernesto University Hospital (HUPE/UERJ), based on radiographic and tomographic imaging, epidemiological data and the routine diagnostic test results. Volunteers in the HC group were recruited based on the absence of any medical conditions, non-smoking status, and lack of comorbidities. Diagnosis of TB was made when at least one of the following criteria was met: (I) positive *M. tuberculosis* culture or Xpert MTB/RIF^®^ assay; (II) acid-fast bacilli (AFB) staining of Mtb from sputum, pleural fluid, or pleural tissue; (III) presence of pleural granuloma with or without caseous necrosis on histopathological analysis; (IV) clinical features or radiological images consistent with PTB and/or PlTB, such as fever, pleuritic chest pain, dyspnea, cough, night sweats, hyporexia, and/or weight loss; (V) exudative pleural effusion with ADA levels > 40 U/L; (VI) complete recovery following a 6-month empirical anti-TB treatment. Participants in the OD group had lymphoma, lung adenocarcinoma, or other neoplasms causing pleural effusions, with diagnoses based on routine diagnostic testing (Table 1).

**TABLE 1:** Baseline characteristics of study population.

Characteristics	HC (*n* = 10)	PTB (*n* = 10)	PlTB (*n* = 18)		OD (*n* = 20)		*P*-value
	Serum (*n* = 10)	Serum (*n* = 10)	Serum* (*n* = 9)	Pleural fluid* (*n* = 9)	Serum* (*n* = 10)	Pleural fluid* (*n* = 10)	
Demographics							
Male, *n* %	40	50	61,1		55		0.779
Age, mean ± SD	34.8 ± 11.9	44.5 ± 12.1	41.1 ± 15.8		60.2 ± 15.7		0.0001
Comorbidities, n %							
Diabetes mellitus	0	10	11.11		5		0.758
Hypertension	0	20	11.11		30		0.164
Pneumoniae	0	0	0		10		0.322
Hepatitis	0	0	0		5		1.000
Clinical							
Diagnostic, *n* %							
Pulmonary TB	N/A	100	0		0		**–**
Pleural TB	N/A	0	100		0		**–**
Adenocarcinoma	N/A	0	0		50		**–**
Other neoplasmas	N/A	0	0		50		**–**
Mycobacterial culture, n %							
Positive	N/A	40	5.5		0		<0.001
AFB, *n* %							
Positive	N/A	30	0		0		0.008
ADA (IU/L)							
Mean ± SD	N/A	N/A	52.4 ± 20.1		11.4 ± 6.5		<0.001
Positive (≥40), *n* %	N/A	N/A	61.11		0		<0.001
LDH (IU/L) in pleural fluid, mean ± SD	N/A	N/A	555 ± 384		515 ± 364		0.761
Histopathology, n %							
Presence of granuloma	N/A	0	50		0		<0.001
Cellular characteristic of the pleural effusion, *n* %	**–**	**–**	**–**		**–**		0.103
Mononuclear	N/A	N/A	81.25		100		**–**
Polimorphonuclear	N/A	N/A	18.75		0		**–**

N/A, not applicable; ADA, adenosine deaminase; LDH, lactate dehydrogenase; AFB, acid-fast bacilli; SD, standard deviation; PTB, pulmonary tuberculosis; PlTB, pleural tuberculosis; HC, healthy controls; OD, other diseases (non-tuberculous pleural effusions). Data are presented as mean ± standard deviation (SD) for continuous variables and as percentages for categorical variables. Continuous variables were analyzed using one-way analysis of variance (ANOVA), followed by Dunnett’s multiple comparisons test for comparisons with the reference group, or an unpaired *t*-test with Welch’s correction for two-group comparisons, as appropriate. Categorical variables were analyzed using Fisher’s exact test. A *p*-value ≤ 0.05 was considered statistically significant. *Unpaired samples.

### Targeted liquid chromatography mass spectrometry (LC-MS)- based metabolomics

Serum and pleural fluid samples were analyzed using the AbsoluteIDQ p180 kit (Biocrates Life Sciences). Samples were randomized, and 10 μL of each sample was added to a 96-well plate together with 10 μL of isotopically labeled internal standards. A seven-point calibration curve (10 μL; three replicates), phosphate-buffered saline (PBS) for blank correction, and three levels of quality controls (QC) were included on each plate. The filter plate was dried under nitrogen, followed by the addition of phenyl isocyanate (PITC) for derivatization. Analytes were extracted using ammonium acetate in methanol (5 mmol/L). The resulting eluate was divided between two plates, one for LC-MS analysis and one for flow-injection analysis (FIA) and further diluted using defined volumes. Solvents used for solution preparation and LC-MS/MS mobile phases included Optima™ LC-MS-grade methanol (MeOH), acetonitrile (ACN), water (H2O), isopropanol (IPA), and formic acid (FA), all obtained from Fisher Scientific. Analysis was undertaken using a Xevo TQ-S Triple Quadrupole Mass Spectrometer coupled to an Acquity UPLC system (Waters Corporation). Chromatographic separation was accomplished using a BEH C18 1.7 μm 2.1 × 75 mm reversed phase column (Waters Acquity) using two mobile phases, solvent A: Water with 0.2% formic acid and solvent B: 100% acetonitrile with 0.2% formic acid using a gradient profile specified in the manufacturer’s protocol. The FIA run utilized methanol with a modifier provided by the manufacturer.

Metabolites were quantified on each plate using a seven-point calibration curve, while additional analytes were semi-quantified using a single-point standard. Metabolite concentrations in each QC level were compared with expected values, and coefficients of variation (CV%) were calculated. Metabolites were excluded if the CV% for QC2 exceeded 30% or if more than 25% of samples fell below the limit of detection, below the lower limit of quantification, above the upper limit of quantification, or outside the acceptable blank range (*n* = 48 were excluded).

### Metabolomic and statistical analysis

Multivariate analysis was performed using principal component analysis (PCA) and orthogonal partial least squares–discriminant analysis (OPLS-DA) in MetaboAnalyst Biomarker Analysis 6.0 (12). Data were log_10_-transformed and Pareto-scaled prior to analysis. PCA was applied to assess overall variance structure (unsupervised), whereas OPLS-DA was used to model class-predictive variation (supervised). Variable Importance in Projection (VIP) scores were extracted to identify metabolites contributing to discrimination between PlTB and comparator groups, with VIP > 1 indicating a significant contribution. Statistical tests including analysis of variance (ANOVA), Dunnett’s multiple comparison test, and unpaired *t*-tests with Welch’s correction were used to independently evaluate model-derived differences. Receiver operating characteristic (ROC) analyses were performed using GraphPad to assess classifier performance. Area under the curve (AUC) values were estimated using logistic regression, and *p*-values were calculated using *t*-tests. AUC values ranged from 0.5 (no discriminative ability) to 1 (perfect discrimination). All statistical tests were interpreted using a significance threshold of *p* ≤ 0.05.

## Results

### Serum metabolite signatures for distinction of PITB from pulmonary TB

**FIGURE 1:**
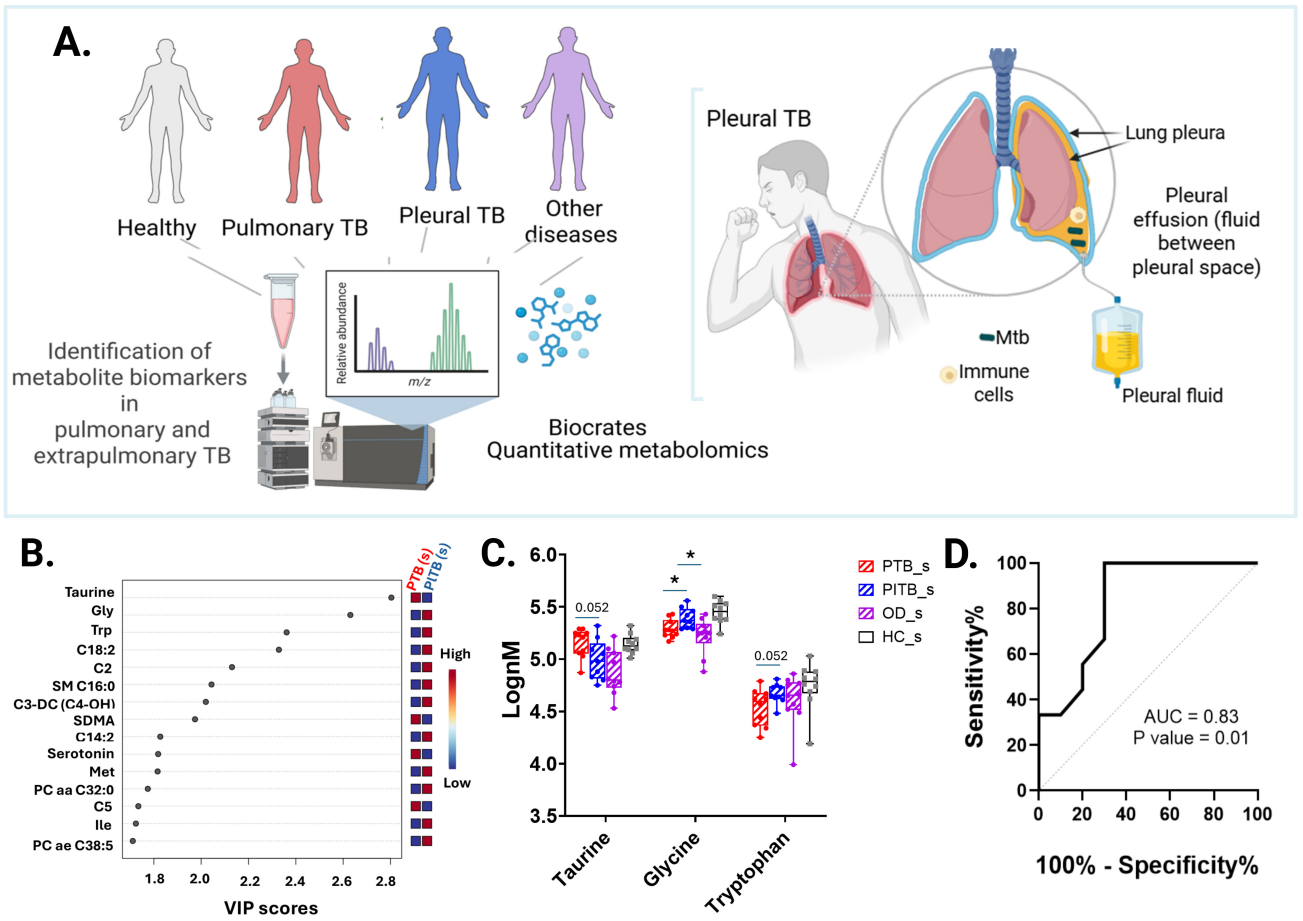
Metabolic classifiers in serum for differentiation of pleural TB. **(A)** Overview of the metabolomics study. The study included four groups: healthy controls (HC), individuals with pulmonary TB (PTB), pleural TB (PlTB) and other diseases (OD). An overview of pleural TB (PlTB) is shown as a subfigure. Serum from all four groups and pleural fluid from PlTB and ODs were collected for this study (see method of technical details). Briefly, serum and pleural fluid samples were processed using Biocrates quantitative metabolomics kit and LC-MS/MS targeted analyses to quantify 180 metabolites. **(B)** VIP score plot showing top 15 serum metabolites that differentiated PlTB from PTB (list of metabolites on Y-axis and scores on X-axis). **(C)** Box plot showing taurine, glycine and tryptophan as the top metabolites that significantly differentiates PlTB from PTB in serum. **(D)** ROC analysis of serum glycine as the top model for separation of PlTB vs. PTB. Graph shows area under the curve (AUC) (95% confidence intervals: 0.67 to 1), and sensitivity and specificity on X- and Y-axis, respectively. Values are mean ± S.D. (*N* = 9–10). *Indicates statistically significant differences calculated using Welch’s *t*-test, *p* ≤ 0.05. PTB (s), serum metabolites measured in PTB group; PlTB (s), serum metabolites measured in PlTB group.

**FIGURE 2:**
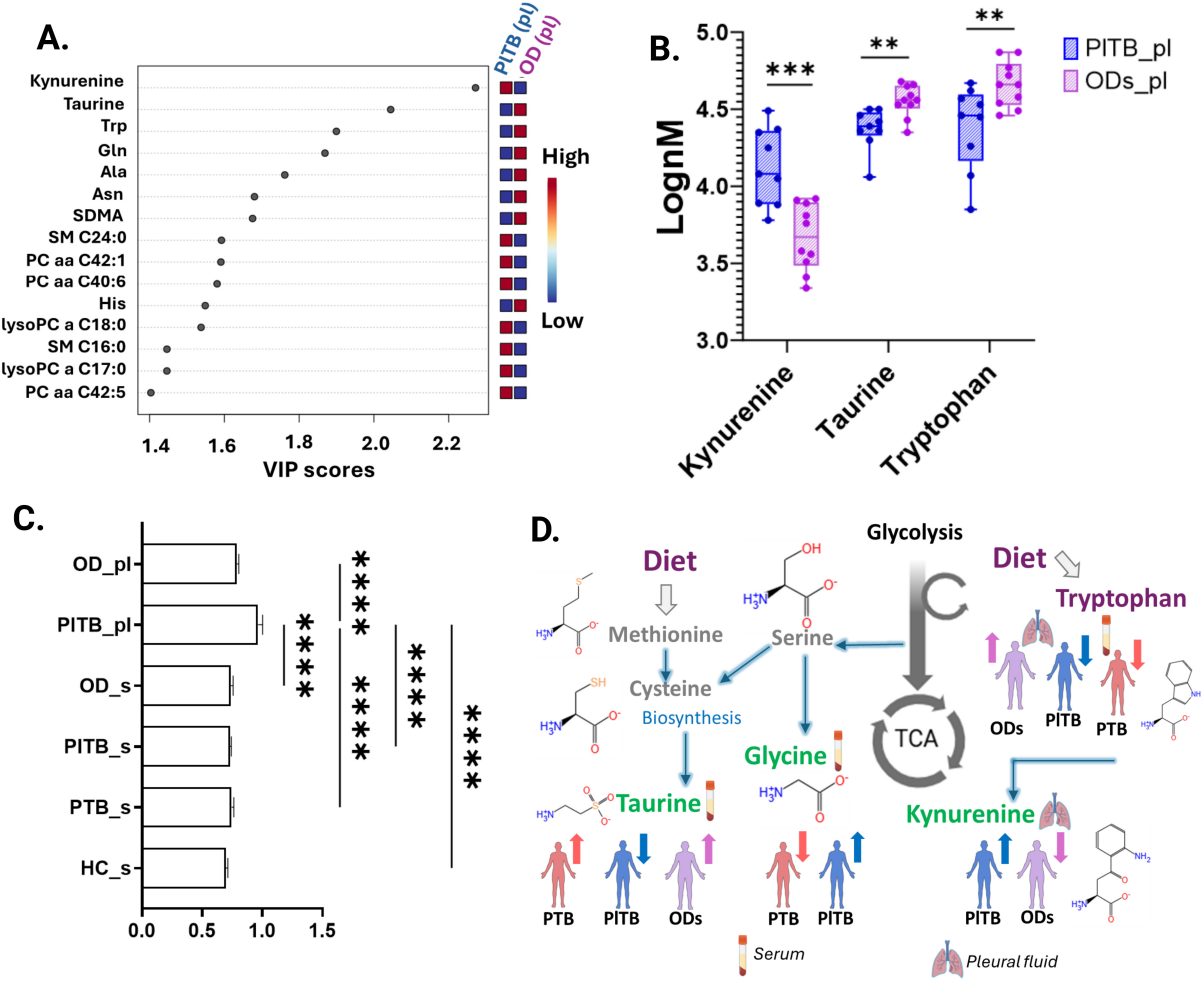
Metabolite features in pleural fluid for differentiation of pleural TB from non-TB pleural effusions in other diseases (ODs). **(A)** VIP score plot showing top 15 pleural fluid metabolites that separated PlTB from ODs. **(B)** Box plot showing kynurenine, taurine, and tryptophan as the top pleural fluid metabolites that significantly differentiates PlTB from ODs. Values are mean ± S.D. (*N* = 9–10). **, ***Indicates statistically significant differences calculated using Welch’s *t*-test, *p* ≤ 0.05 and *p* ≤ 0.005. **(C)** Kynurenine to tryptophan, K/T ratio quantified across six groups. Serum measurements are indicated as HC_s, PTB_s, PlTB_s, OD_s and pleural fluid levels as PITB_pl and OD_pl. One-way Anova and Tukey’s multiple comparisons test was used to compare different groups; *indicates statistically significant differences. HC, healthy control; PTB, pulmonary TB; PlTB (s), serum from pleural TB; PlTB_pl, pleural fluid from pleural TB. **(D)** A summary figure showing the key metabolite classifiers and their associated metabolic pathways. Methionine derived from diet is the precursor for taurine that separated PlTB from PTB and ODs. Glycine is derived from serine which is a non-essential amino acid synthesized from glycolysis. Glycine is higher in PTB and lower in PlTB. Tryptophan is procured from diet and degraded into kynurenine. Both tryptophan and kynurenine are markers differentiating PlTB from PTB and ODs. PlTB (pl), pleural fluid metabolites measured in PlTB group; OD (pl), pleural fluid metabolites measured in OD group.

To define metabolic features specific to PITB, we performed targeted metabolomic profiling of serum from HC, PTB, PITB, and pleural fluid from PITB and OD groups. Serum samples were available for all four groups, whereas pleural fluid could only be collected from individuals with pleural effusions: the PITB and OD groups. To ensure biologically meaningful comparisons, serum metabolite analyses were conducted across PITB, PTB, HC, and OD (Figure 1), while pleural fluid metabolomics were restricted to PITB and OD (Figure 2). This experimental design was used to minimize any artifacts arising from mismatched sample types and enabled identification of matrix-specific metabolic signatures.

Demographic characteristics were comparable across groups, with no significant differences in the age and gender composition of participants across the four groups, except for OD group with a higher mean age of 60.2 years. The higher age in the OD group reflects the age-associated pathophysiology of disease, as older adults constituted a significant proportion of individuals with cancer and malignant pleural effusions. The demographic and clinical features of the cohort in Table 1 shows that the metabolite features specific to PITB identified in our study for are a direct consequence of TB infection.

The clinical sample requirement was minimized by using a highly sensitive assay requiring only 10 μL of sample, which is 1/10^th^–100^th^ of the volume used in other metabolomic studies (13, 14). This reduced sample volume minimizes the risk of complications associated with pleural fluid collection in patients. Using a high-throughput LC-MS/MS 96-well plate metabolomics we quantified 140 metabolites (14 acylcarnitines, 21 amino acids, 10 biogenic amines, 81 phosphatidyl cholines, and 14 sphingolipids) across all four groups (Supplementary Table 1, Supplementary File 1).

One of our primary objectives was to identify serum-based metabolic signatures that robustly distinguished PITB from PTB. OPLS-DA modeling demonstrated a clear and reproducible separation between the two groups, driven by 15 metabolites with VIP scores ≥ 1.5 (Figure 1B). Among these, taurine, glycine, and tryptophan emerged as the top discriminatory serum metabolites. Taurine levels were significantly reduced in PITB, whereas glycine and tryptophan were elevated relative to PTB (Figure 1C), indicating a distinct serum metabolic profile associated with pleural TB. ROC analysis further confirmed glycine as the top serum classifier for PITB versus PTB (AUC = 0.83; Figure 1D). Glycine levels were also higher in PITB compared with OD, but they did not differ significantly between PITB and HC (baseline control), demonstrating that glycine’s serum profile value is specific to PITB. Also, methionine sulfoxide (Met-SO) and histidine, and not taurine, glycine, or tryptophan were the top serum metabolites separating PITB from HC (Supplementary Figure 1). In summary, these analyses confirm that glycine is an important PITB-specific metabolic discriminator in serum, highlighting its biological and diagnostic relevance to distinguish PITB from PTB.

### Pleural fluid metabolic markers for distinction of PITB from non-TB pleural effusions

We compared pleural fluid metabolites between PITB and OD to identify pleural fluid-specific metabolic signatures, as pleural fluid is available only from individuals with effusions and therefore cannot be obtained from PTB or healthy controls. Multivariate OPLS-DA and VIP analyses identified kynurenine, taurine, and tryptophan as the top pleural-fluid metabolites distinguishing PITB from OD (Figure 2A). ROC analysis further confirmed their diagnostic strength, with all three metabolites achieving AUC values ≥ 0.8, indicating robust discriminatory performance (Supplementary Figure 2). PITB pleural fluid exhibited significantly elevated kynurenine levels, and reduced taurine and tryptophan compared to OD (Figure 2B). When examined across all study groups, PITB consistently showed the lowest pleural fluid taurine and tryptophan levels and the highest kynurenine levels, establishing these metabolites as distinct classifiers for PITB in pleural fluid (Supplementary Figure 3). Indoleamine 2,3-dioxygenase-1 (IDO1) catalyzes the conversion of tryptophan to kynurenine and plays a central role in immune activation. Here we further examined the kynurenine/tryptophan ratio across all study groups. PITB demonstrated the highest ratio of all groups (Figure 2C), providing the first experimental evidence that enhanced tryptophan catabolism is a defining metabolic feature of pleural TB. This pathway is well documented in pulmonary TB and appears more pronounced in PITB highlighting it as a potential biomarker with strong diagnostic potential.

## Discussion

The diagnosis of pleural TB remains challenging due to the low bacterial burden at the site of infection, and the compartmentalized nature of immune responses. As a result, accurate diagnosis often requires the use of invasive procedures such as thoracentesis or pleural biopsies, which complicate disease management and can restrict accessibility in resource-limited settings (5, 6). In this study we optimized a 96-well plate-based LC-MS/MS metabolomics assay to identify metabolic features implicated in pleural TB. This high-throughput approach enabled robust profiling of both serum and pleural fluid metabolites, facilitating the discovery of candidate biomarkers with the potential to improve diagnostic accuracy for pleural TB.

The comparison of serum metabolite profiles between HC and PITB groups identified Met-SO and HIS as the top candidates to distinguish patients with pleural TB from healthy individuals. Both Met-SO and HIS were reduced indicating changes in the metabolism of these two amino acids in pleural TB. Met-SO, derived from the oxidation of methionine, is a clinical biomarker of oxidative stress in the serum proteins. In our study, methionine levels did not differ significantly between HC and PITB groups. Interestingly, Met-SO levels were reduced in PlTB compared to HC, which given previous reports of elevated Met-SO in pulmonary TB, attributed to infection and inflammation associated oxidative stress (15, 16). A plausible explanation for this reduction is increased activity of methionine sulfoxide reductases, which may act to preserve redox balance and limit oxidative damage during pleural TB infection. Alternatively, further oxidation of Met-SO to methionine sulfone, which is an irreversible reaction could contribute to the reduced Met-SO levels in PlTB samples. However, our LC-MS/MS workflow did not capture methionine sulfone, limiting experimental evidence to support this hypothesis. Genes involved in methionine oxidation and reduction are increasingly recognized as relevant in TB, and methionine sulfoxide reductases (MsrA and MsrB) have recently attracted attention as potential diagnostic targets. However, their precise roles in TB pathogenesis and their utility as diagnostic biomarkers needs to be established. Previous metabolomics study identified reduced levels of HIS as a marker to distinguish pulmonary TB from healthy controls and highlighted its involvement in regulating blood coagulation pathways in TB patients (17, 18). Here we have shown that HIS is also an important metabolic marker for pleural TB.

Taurine, glycine, tryptophan and kynurenine were identified as the top metabolites in differentiating pleural TB from pulmonary TB and other non-TB pleural effusions. Taurine metabolism plays a key role in resolving inflammation associated with infections, sepsis, chronic conditions such as cancer, and inflammatory diseases including arthritis and multiple sclerosis (19, 20). Taurine metabolism results in the formation of taurine chloramine, which has both antimicrobial and anti-inflammatory properties (19). Taurine has been linked to the induction of trained immunity in the context of TB, particularly in individuals who received BCG vaccination (21).

Glycine, a non-essential amino acid produced primarily from glycolytic metabolism is an important metabolic feature for PITB (Figure 2D). The glycine levels observed in PlTB patients contrasted with that observed in Mtb-infected human macrophages *in vitro*, where infected macrophages exhibited significantly reduced glycine fluxes compared to the uninfected macrophages (22). This discrepancy highlights that *in vitro* macrophage infection models may be relevant for studying pulmonary TB but do not fully capture the metabolic features of pleural TB. Moreover, previous studies have shown that plasma glycine levels were significantly lower in individuals with TB and diabetes mellitus co-morbidities (17). Taken together, our findings and other studies highlight glycine as a robust metabolic marker capable of differentiating PlTB from PTB, as well as PlTB from TB with co-morbidities such as diabetes, thereby reinforcing its potential diagnostic utility across diverse clinical contexts (17, 23, 24).

Previous metabolomics studies employing gas chromatography-mass spectrometry (GC-MS) and LC-MS metabolomics did not assess tryptophan and kynurenine levels in PlTB (9, 14). Tryptophan and kynurenine are immunomodulatory metabolites that drive inflammatory responses and T cell responses in TB, as well as in other inflammatory conditions and diseases such as multiple sclerosis and cancer (13, 25, 26).

Tryptophan, an essential amino acid, plays a critical role in TB pathogenesis (Figure 2D). We and others have shown that tryptophan levels are reduced in PTB and PlTB and its catabolism to kynurenine is elevated in PTB and PlTB compared to the healthy individuals (13, 17). The increased biosynthesis of kynurenine from tryptophan played an important role in maintaining cellular energetics through de novo synthesis of pyridine nucleotide NAD+, which is essential for cellular metabolism and function (13, 26). Importantly, the kynurenine/tryptophan ratio previously established as a key classifier for PTB was highest in pleural fluid from PlTB patients in our study. This finding provides new evidence that enhanced tryptophan catabolism is a defining metabolic feature of PlTB and highlights the kynurenine/tryptophan ratio as a promising candidate biomarker for differentiating PlTB from PTB. Changes in the kynurenine/tryptophan ratio and/or kynurenine upregulation have also recently been shown to be conserved in diverse non-human in vivo models of TB, including mice, infant rhesus macaques, and zebrafish larvae (27–29). The conservation of the host kynurenine/tryptophan profile in response to mycobacterial infection across species suggests that this biomarker may have utility for investigating Mtb complex infections in non-human hosts, supporting a One Health framework for disease surveillance and management.

## Conclusion

This pilot study identified novel metabolite features that clearly distinguish PlTB from PTB and OD. We also developed a 96-well plate–based LC-MS/MS assay with substantially enhanced sensitivity, capable of detecting metabolites from as little as 10 μL of sample. This platform offers a highly sensitive and accessible approach for biomarker analysis and can be readily implemented in a wide range of analytical laboratory settings. By reducing the sample volume required for metabolomic analysis, the assay also helps minimize the risk of complications associated with pleural fluid collection. Our findings highlight glycine, tryptophan, kynurenine, and taurine as key metabolic signatures for differentiating PlTB, representing promising candidates for future biomarker development.

## Limitations

The relatively small number of participants in each group may have limited the number of classifiers identified. The metabolite features reported here require validation in larger, independent cohorts. Despite these limitations, this study provides clinically meaningful metabolic signatures that supports improved differentiation of extrapulmonary TB.

## Data Availability

The original contributions presented in this study are included in the article/Supplementary material, further inquiries can be directed to the corresponding author.

## Supplementary material

The raw mass spec data sets are available in Mendeley datarepository: BORAH SLATER, KHUSHBOO; May-Lewis, Holly (2026), “Metabolic signatures in serum and pleural fluid of individuals with tuberculosis pleural effusion.” Mendeley Data, V1, doi: 10.17632/z86dk5wknn.1.
