# Supplementary material for: Metabolic biomarkers differentiate extrapulmonary tuberculosis from pulmonary TB and non-TB pleural effusions
Source: Front Med (Lausanne). 2026 Jun 11;13:1793040. doi: 10.3389/fmed.2026.1793040 (PMC13293869; doi:10.3389/fmed.2026.1793040)
Supplement: Supplementary file 1 [file Supplementary_file_1.docx]

Title: **Metabolic Biomarkers Differentiate Extrapulmonary Tuberculosis from Pulmonary TB and Non‑TB Pleural Effusions**

Running title: **Metabolic signatures for pleural tuberculosis**

**Authors:** Holly May-Lewis^1^, Michael James Perret^2^, Ye Xu^3^, Roberto Stefan Almeida Ribeiro^4^, Raquel da Silva Correa^4^, Thiago Thomaz Mafort^5^, Ana Paula Santos^5^, Rogério Rufino^5^, Luciana Rodrigues^4^, Khushboo Borah Slater^2*^

^1^School of Biosciences, University of Surrey, Guildford, United Kingdom

^2^Department of Biosciences, Faculty of Health and Life Sciences, University of Exeter, EX4 4QD

^3^Department of Clinical Laboratory, Shanghai Ninth People’s Hospital, Shanghai Jiao Tong University School of Medicine, Shanghai, China

^4^Department of Pathology and Laboratories, Laboratory of Immunopathology, Medical Sciences Faculty (FCM), Rio de Janeiro State University (UERJ), Rio de Janeiro, RJ, Brazil.

^5^Department of Pulmonary Care, Pedro Ernesto University Hospital (HUPE), UERJ, Rio de Janeiro, RJ, Brazil.

^*^Corresponding author email: [k.borah-slater@exeter.ac.uk](mailto:k.borah-slater@exeter.ac.uk)

Supplementary Figure 1


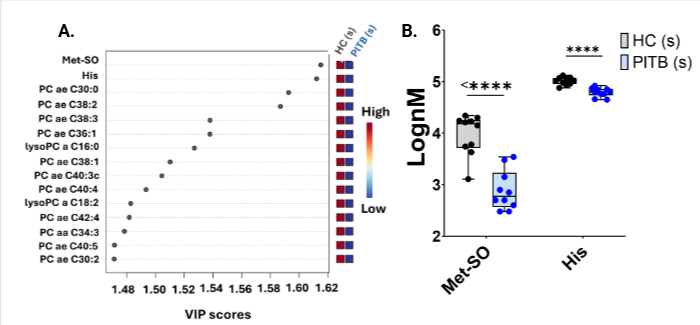


Supplementary Figure. 1. **Metabolite features in serum for differentiation of pleural TB from healthy controls (HC)**. A**.** VIP scores showing 15 ranked **serum** metabolites that separated PlTB from HC. B**.** Box plot showing methionine sulfoxide (Met-SO) abundances in PlTB vs. HC. Values are mean ± S.D. (N=9-10). * Indicates statistically significant differences calculated using Welch’s t-tests, p ≤0.05. HC: healthy control; PlTB: pleural TB.

Supplementary Figure 2


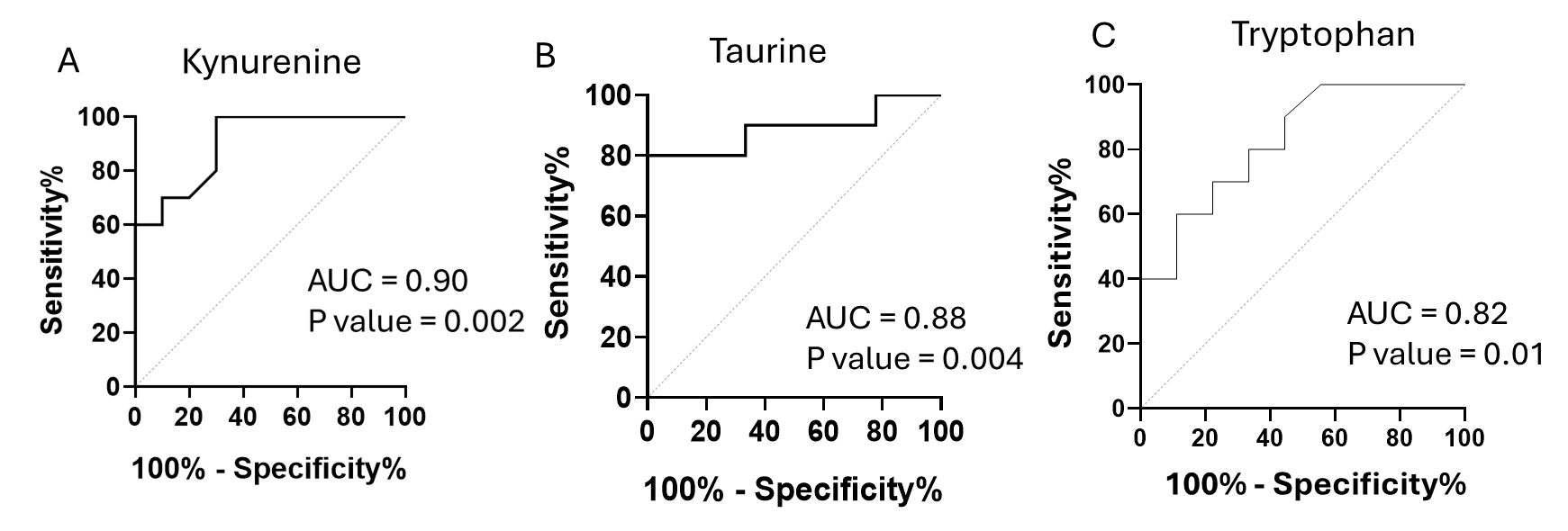


Supplementary Figure 2. **ROC analysis of kynurenine, taurine and tryptophan as metabolic classifiers in pleural fluid of PlTB vs PTB**. ROC analysis shows AUC for A) kynurenine (95% CI:0.77-1.0) B) taurine (95% CI:0.75-1.0) C) tryptophan (95% CI: 0.67-1.0). P values were calculated using Wilson/Brown tests. The sensitivity and specificity are shown on the X- and Y-axis respectively. Abbreviations: PlTB: pleural TB; OD: non-TB pleural effusions.

Supplementary Figure 3


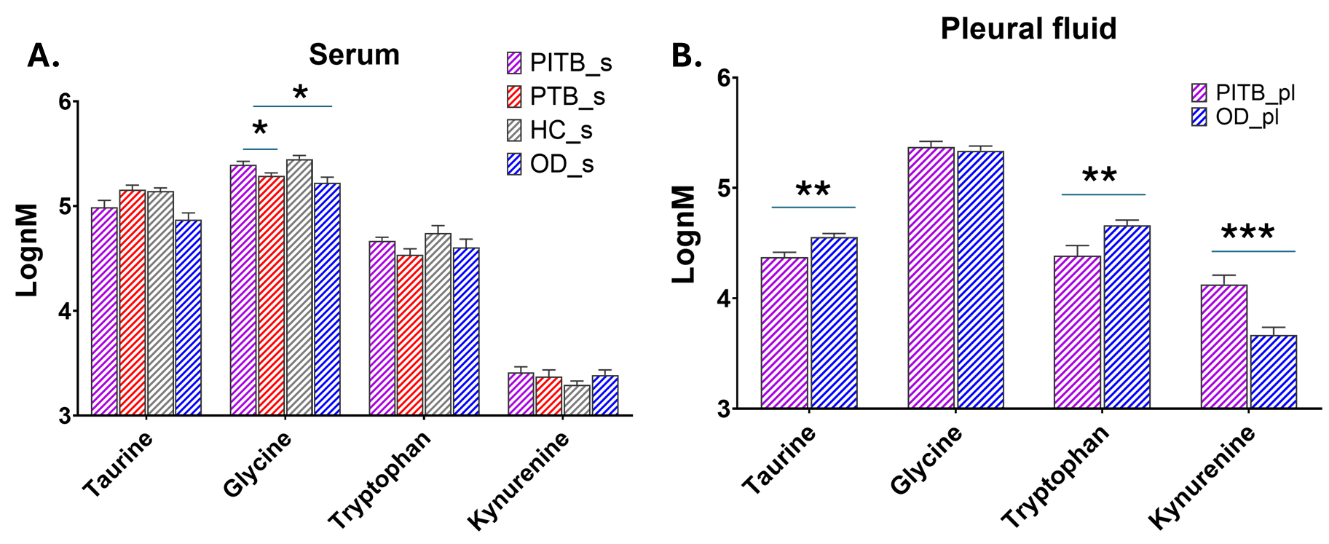


Supplementary Figure 3. **Serum and pleural fluid metabolite classifier profiles**. Taurine, glycine, tryptophan and kynurenine profiles in A. serum and B. pleural fluid samples. Groups analysed for serum samples include HC: health control; PTB: pulmonary TB; PITB: pleural TB; OD: other diseases (non-TB pleural effusions). Pleural fluid samples were analysed for PITB and OD. Statistical significance between PITB_s or PITB_pl versus other groups was assessed using Welch’s t-test; * indicates statistically significant differences between two groups (P≤0.05).
