# Supplementary material for: Metabolic biomarkers differentiate extrapulmonary tuberculosis from pulmonary TB and non-TB pleural effusions
Source: Front Med (Lausanne). 2026 Jun 11;13:1793040. doi: 10.3389/fmed.2026.1793040 (PMC13293869; doi:10.3389/fmed.2026.1793040)
Supplement: Supplementary file 2 [file Table_1.docx]

Table S1. List of metabolites identified using AbsoluteIDQ® p180 kit (http://biocrates.com/)

| Amino acids (21) | | | | |
| --- | --- | --- | --- | --- |
| Ala | | Alanine | Lys | Lysine |
| Arg | | Arginine | Met | Methionine |
| Asn | | Asparagine | Orn | Ornithine |
| Asp | | Aspartate | Phe | Phenylalanine |
| Cit | | Citrulline | Pro | Proline |
| Glu | | Glutamate | Ser | Serine |
| Gln | | Glutamine | Thr | Threonine |
| Gly | | Glycine | Trp | Tryptophan |
| His | | Histidine | Tyr | Tyrosine |
| Ile | | Isoleucine | Val | Valine |
| Leu | Leucine | |  | |

| Biogenic amines (21) | | | | |
| --- | --- | --- | --- | --- |
| Ac-Orn | | Acetylornithine | Met-SO | Methionine sulfoxide |
| alpha-AAA | | alpha-Aminoadipic acid | Nitro-Tyr1 | Nitrotyrosine |
| ADMA | | Asymmetric dimethylarginine | PEA | Phenylethylamine |
| Carnosine | | Carnosine | Putrescine | Putrescine |
| Creatinine | | Creatinine | Sarcosine | Sarcosine |
| DOPA | | Dihydroxyphenylalanine | Serotonin | Serotonin |
| Dopamine | | Dopamine | Spermidine | Spermidine |
| Histamine | | Histamine | Spermine | Spermine |
| c4-OH-Pro | | cis-4-Hydroxyproline | SDMA | Symmetric dimethylarginine |
| t4-OH-Pro | | trans-4-Hydroxyproline | Taurine | Taurine |
| Kynurenine | Kynurenine | |  | |

| Monosaccharides (1) | |
| --- | --- |
| H1 | Hexoses (including glucose) |

| Acylcarnitines (40) | | | |
| --- | --- | --- | --- |
| C0 | Carnitine | C5-DC (C6-OH) | Glutarylcarnitine (Hydroxyhexanoylcarnitine) |
| C2 | Acetylcarnitine | C5-M-DC | Methylglutarylcarnitine |
| C3 | Propionylcarnitine | C5-OH (C3-DC-M) | Hydroxyvalerylcarnitine (Methylmalonylcarnitine) |
| C3-OH | Hydroxypropionylcarnitine | C5:1 | Tiglylcarnitine |
| C3:1 | Propenoylcarnitine | C5:1-DC | Glutaconylcarnitine |
| C4 | Butyrylcarnitine | C6 (C4:1-DC) | Hexanoylcarnitine (Fumarylcarnitine) |
| C4-OH (C3-DC) | Hydroxybutyrylcarnitine (Malonylcarnitine) | C6:1 | Hexenoylcarnitine |
| C4:1 | Butenylcarnitine | C7-DC | Pimeloylcarnitine |
| C5 | Valerylcarnitine | C8 | Octanoylcarnitine |

| Acylcarnitines (continued) | | | |
| --- | --- | --- | --- |
| C9 | Nonaylcarnitine | C14:2-OH | Hydroxytetradecadienylcarnitine |
| C10 | Decanoylcarnitine | C16 | Hexadecanoylcarnitine |
| C10:1 | Decenoylcarnitine | C16-OH | Hydroxyhexadecanoylcarnitine |
| C10:2 | Decadienylcarnitine | C16:1 | Hexadecenoylcarnitine |
| C12 | Dodecanoylcarnitine | C16:1-OH | Hydroxyhexadecenoylcarnitine |
| C12-DC | Dodecanedioylcarnitine | C16:2 | Hexadecadienylcarnitine |
| C12:1 | Dodecenoylcarnitine | C16:2-OH | Hydroxyhexadecadienylcarnitine |
| C14 | Tetradecanoylcarnitine | C18 | Octadecanoylcarnitine |
| C14:1 | Tetradecenoylcarnitine | C18:1 | Octadecenoylcarnitine |
| C14:1-OH | Hydroxytetradecenoylcarnitine | C18:1-OH | Hydroxyoctadecenoylcarnitine |
| C14:2 | Tetradecadienylcarnitine | C18:2 | Octadecadienylcarnitine |

| Glycerophospholipids (90) | | | | | |
| --- | --- | --- | --- | --- | --- |
| lysoPC a C14:0 | | PC aa C34:1 | | PC aa C42:0 | PC ae C38:2 |
| lysoPC a C16:0 | | PC aa C34:2 | | PC aa C42:1 | PC ae C38:3 |
| lysoPC a C16:1 | | PC aa C34:3 | | PC aa C42:2 | PC ae C38:4 |
| lysoPC a C17:0 | | PC aa C34:4 | | PC aa C42:4 | PC ae C38:5 |
| lysoPC a C18:0 | | PC aa C36:0 | | PC aa C42:5 | PC ae C38:6 |
| lysoPC a C18:1 | | PC aa C36:1 | | PC aa C42:6 | PC ae C40:1 |
| lysoPC a C18:2 | | PC aa C36:2 | | PC ae C30:0 | PC ae C40:2 |
| lysoPC a C20:3 | | PC aa C36:3 | | PC ae C30:1 | PC ae C40:3 |
| lysoPC a C20:4 | | PC aa C36:4 | | PC ae C30:2 | PC ae C40:4 |
| lysoPC a C24:0 | | PC aa C36:5 | | PC ae C32:1 | PC ae C40:5 |
| lysoPC a C26:0 | | PC aa C36:6 | | PC ae C32:2 | PC ae C40:6 |
| lysoPC a C26:1 | | PC aa C38:0 | | PC ae C34:0 | PC ae C42:0 |
| lysoPC a C28:0 | | PC aa C38:12 | | PC ae C34:1 | PC ae C42:1 |
| lysoPC a C28:1 | | PC aa C38:3 | | PC ae C34:2 | PC ae C42:2 |
| PC aa C24:0 | | PC aa C38:4 | | PC ae C34:3 | PC ae C42:3 |
| PC aa C26:0 | | PC aa C38:5 | | PC ae C36:0 | PC ae C42:4 |
| PC aa C28:1 | | PC aa C38:6 | | PC ae C36:1 | PC ae C42:5 |
| PC aa C30:0 | | PC aa C40:1 | | PC ae C36:2 | PC ae C44:3 |
| PC aa C30:22 | | PC aa C40:2 | | PC ae C36:3 | PC ae C44:4 |
| PC aa C32:0 | | PC aa C40:3 | | PC ae C36:4 | PC ae C44:5 |
| PC aa C32:1 | | PC aa C40:4 | | PC ae C36:5 | PC ae C44:6 |
| PC aa C32:2 | PC aa C40:5 | | PC ae C38:0 | | |
| PC aa C32:3 | PC aa C40:6 | | PC ae C38:1 | | |

| Sphingomyelins (15) | | | | | |
| --- | --- | --- | --- | --- | --- |
| SM (OH) C14:1 | SM C18:0 | | | SM (OH) C22:2 | SM (OH) C24:1 |
| SM C16:0 | SM C18:1 | | | SM C22:32 | SM C26:0 |
| SM C16:1 | SM C20:2 | | | SM C24:0 | SM C26:1 |
| SM (OH) C16:1 | | SM (OH) C22:1 | SM C24:1 | | |
